# Evolution of *Mutator* transposable elements across eukaryotic diversity

**DOI:** 10.1186/s13100-019-0153-8

**Published:** 2019-03-21

**Authors:** Mathilde Dupeyron, Kumar S. Singh, Chris Bass, Alexander Hayward

**Affiliations:** Centre for Ecology and Conservation, University of Exeter, Penryn Campus, Penryn, Cornwall, TR10 9FE UK

**Keywords:** MULE, MULEs, MuDR, Transposon, Horizontal transmission, Horizontal transfer, Phylogenetic analysis

## Abstract

**Background:**

*Mutator*-like elements (MULEs) are a significant superfamily of DNA transposons on account of their: (i) great transpositional activity and propensity for insertion in or near gene sequences, (ii) their consequent high mutagenic capacity, and, (iii) their tendency to acquire host gene fragments. Consequently, MULEs are important genetic tools and represent a key study system for research into host-transposon interactions. Yet, while several studies have focused on the impacts of MULEs on crop and fungus genomes, their evolution remains poorly explored.

**Results:**

We perform comprehensive bioinformatic and phylogenetic analyses to address currently available MULE diversity and reconstruct evolution for the group. For this, we mine MULEs from online databases, and combine search results with available transposase sequences retrieved from previously published studies. Our analyses uncover two entirely new MULE clades that contain elements almost entirely restricted to arthropod hosts, considerably expanding the set of MULEs known from this group, suggesting that many additional MULEs may await discovery from further arthropod genomes. In several cases, close relationships occur between MULEs recovered from distantly related host organisms, suggesting that horizontal transfer events may have played an important role in the evolution of the group. However, it is apparent that MULEs from plants remain separate from MULEs identified from other host groups. MULE structure varies considerably across phylogeny, and TIR length is shown to vary greatly both within and between MULE groups. Our phylogeny suggests that MULE diversity is clustered in well-supported groups, typically according to host taxonomy. With reference to this, we make suggestions on how MULE diversity can be partitioned to provide a robust taxonomic framework.

**Conclusions:**

Our study represents a considerable advance in the understanding of MULE diversity, host range and evolution, and provides a taxonomic framework for the classification of further MULE elements that await discovery. Our findings also raise a number of questions relating to MULE biology, suggesting that this group will provide a rich avenue for future study.

**Electronic supplementary material:**

The online version of this article (10.1186/s13100-019-0153-8) contains supplementary material, which is available to authorized users.

## Introduction

*Mutator* transposable elements (TEs) are among the most mutagenic transposons known, due to their very high rates of transposition and their bias for inserting near or close to genes [1, 2]. The original *Robertson’s Mutator* element (*MuDR*, ‘Mutator Don Robertson’) was described in maize, where *Mutator* lines can display mutation frequencies ~ 50 times the background rate of spontaneous mutation [3, 4]. Given these qualities, the *Mutator* system has a long history of usage in the field of genetic engineering in both forward and reverse genetic screening, and has played an important role in shaping our understanding of host-transposon co-evolutionary interactions [2].

Members of the *Mutator* DNA transposon superfamily are termed ‘MULEs’ (*Mutator*-like elements) [5]. The structure of MULEs resembles classic “cut-and-paste” DNA TEs, with Terminal Inverted Repeats (TIRs) at each end enclosing a transposase domain containing a catalytic DDE motif, and often an additional zinc finger DNA-binding motif. However, MULE TIRs are typically considerably longer than those present in other DNA TE superfamilies, commonly being hundreds of base pairs in length. Meanwhile, some families of MULE appear to lack TIRs completely (non-TIR MULEs) [6]. A further characteristic of MULEs is the length of their target site duplications (TSDs), which are short direct repeats created following transposition, that occur immediately outside of the TIRs and are 8-11 bp in MULE elements [5].

An interesting feature of several MULEs is their acquisition of one or more additional open reading frames (ORFs). The first complete MULE element described in maize contained two ORFs: *mudrA*, encoding the transposase, and *mudrB*, encoding a protein of unknown function that is apparently required for integration into the maize genome [7, 8]. Other classes of MULE may also contain additional ORFs, such as the *mutB* ORF with a DNA binding role in the yeast *Mutyl* element that occurs in the same orientation as the transposase ORF [9], or the *vanB* and *vanC* ORFs in the arabidopsis *Hiun* element, which are divergent proteins with unknown and anti-silencing functions, respectively [10]. It is often assumed that additional ORFs originate from the host genome, as has been demonstrated in rice and several other plant genomes [6, 11]. In plant genomes there are large numbers of non-autonomous *Mutator* elements known collectively as ‘*Pack-MULEs’,* that have lost their transposase genes and instead carry fragments of host genes [12]*.* Additionally, there is evidence that several MULEs have undergone molecular domestication and become exapted by host genomes. For example, MULE-derived genes act as transcription factors that modulate the light response in Arabidopsis [13, 14], while the MULE-derived *MUSTANG* genes, which are present in all flowering plants, are involved in diverse processes including flowering, growth and reproduction, and may have played an important role in early angiosperm evolution [15].

MULEs are best known from plant genomes, and especially maize [7], but they also occur in a wide range of eukaryotic genomes [2]. For example, over the last 15 years, studies have described MULEs from diverse hosts, including several ascomycete species [16], the yeast *Yarrowia lipolytica* [9], the Oomycete genus *Phytophthora* [17]*,* amoeba from the genus *Entamoeba* [18], the excavate *Trichomonas vaginalis* [19], diatoms [20], the mosquito *Aedes aegypti* [21], and the trematode *Schistosoma* [22]. However, MULEs were detected in the animal kingdom only recently [23, 24], and in general very little remains known about MULEs from metazoan genomes [21, 22].

In common with other DNA transposons, MULEs contain a DDE catalytic domain with structural features in common with other Class II TEs and the integrase domain of Class I LTR-retrotransposons [25]. The DDE domain is typically more highly conserved than other regions, and DDE sequences are often employed in phylogenetic analyses [26]. Analyses across DNA TE diversity utilising the DDE domain have indicated that MULEs share the closest relationship to *P* and *hAT* elements [26, 27]. Several phylogenetic analyses have also attempted to reconstruct evolution within the MULE superfamily [16, 18, 22, 24]. However, the most recent consideration of MULE evolution was in 2011, where the transposase amino acid sequences of just 39 MULE elements from 26 host genomes resulted in the division of the included sequences into 6 families (MuDR, TvCaMULE, *Curupira*, EMULE, *Hop*/*Jittery*, and *Phantom*) [22]. Given the recent accumulation of genome sequencing data, we revisit the question of MULE evolution. We perform bioinformatic analyses to mine new members of the MULE superfamily, and phylogenetic analyses to update and further investigate the evolutionary history of this important group of DNA TEs.

## Results and discussion

Using a series of genomic database searches, we recovered 1631 autonomous *Mutator*-like elements (MULEs) present in the genomes of hosts from 178 species, across 6 major groupings of eukaryotic life (animals, fungi, amoeba, stramenopiles, parabasalids and plants). Based on the results of our phylogenetic analyses (Additional file 1), and the use of the ClusterPicker pipeline [28], we divided MULE diversity into 50 major lineages, according to the fixed criteria of strict monophyly, clade statistical support, and the genetic distance between sequences. During our database searches, we detected several new groups of MULE elements, and a considerable number of new elements from invertebrate host genomes. We find that TE structure varies considerably across MULE diversity, with elements differing in the possession of additional zinc finger domains and DNA inserts, and varying greatly in TIR length (14-500 bp) and overall element length. Below we discuss our results, and the insights they provide into MULE evolution, host interactions, and MULE taxonomy.

### Two novel MULE clades from invertebrate host genomes

We identify two new MULE clades that we name *Ghost* and *Spectre* which contain 16 and 53 novel MULE sequences respectively, from the genomes of host species in 15 insect and arachnid genera (see Groups 7 and 8, Additional file 1). One of these new clades, *Ghost*, also includes four sequences from cnidarian genomes. All sequences in the *Ghost* and *Spectre* clades were recovered by BLAST searches, using the transposase sequence of a newly identified *Spectre* element as a query, except the 3 cnidarian sequences that were recovered by PSI-BLAST searches using the transposase sequence of MuDR-1_NV (see Methods).

**Fig. 1:**
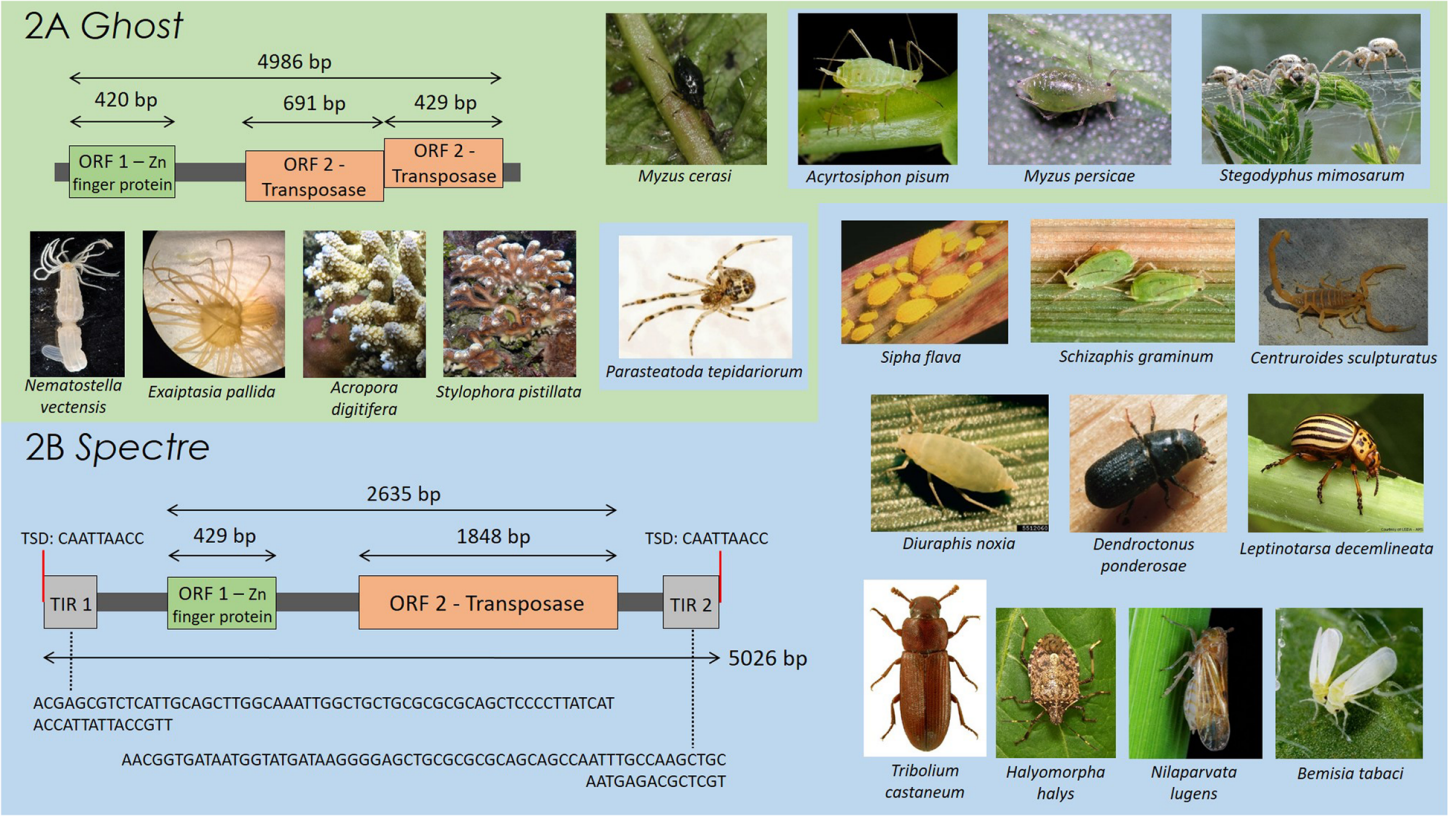
Schematics of the newly identified *Ghost* and *Spectre* elements, with pictures illustrating their respective host ranges as elucidated in this study. 2A) Structure of *Ghost*. 2B) Structure of *Spectre*. TIR: terminal inverted repeat, ORF: open reading frame; bp: base pair; Zn: zinc finger domain; TSD: target site duplication

The *Ghost* clade contains sequences mined from the genomes of the following aphid and spider species: pea aphid *Acyrtosiphon pisum*, green peach aphid *Myzus persicae*, black cherry aphid *Myzus cerasi*, common house spider *Parasteatoda tepidariorum*, and African velvet social spider *Stegodyphus mimosarum*. Since the date of the most recent common ancestor of insects and arachnids is very deep (~ 500-600MYA) [29], and the most recent common ancestor of arthropods and cnidarians (i.e. Protostomia and Deuterostomia) is considerably more ancient, this host distribution pattern implies that at least one horizontal transfer event of *Ghost* elements has occurred within the clade, and across major branches of host diversity. Further, given that *Ghost* spider MULEs (from *S. mimosarum* and *P. tepidariorum*) are nested within a *Ghost* clade containing 11 aphid MULEs, it is likely that there have been further horizontral transmission events between arachnids and insects. Aphids and spiders occupy a shared environment and have a close ecological relationship, and potential horizontal transmission events may have occurred via a mechanism through predation for example [30–32]). A transmission route linking echinoderms and arthropods is less obvious, however, the relatively short branch length between MULEs from these taxa is otherwise hard to explain (Additional file 1). MULEs from additional host taxa that provide a more obvious transmission route may be identified in the future. No complete *Ghost* elements were recovered, and most elements in the clade were represented by a DDE domain only. No TIRs were detected, and only three elements contain a zinc finger motif. The most complete *Ghost* element originates from the genome of the black cherry aphid (*M. cerasi*), and is annotated in Fig. 1a. This element contains two open reading frames: a 420 bp ORF containing a RING zinc finger domain, and a truncated 1120 bp ORF containing the transposase. Despite its truncated transposase ORF, the DDE domain does not contain stop codons and aligns well with other sequences employed in phylogenetic analyses. However, neither TIRs nor TSDs for this element could be identified. It is possible that this apparent lack of TIRs and TSDs may result from sequencing errors, since the *M. cerasi* genome sequence is still provisional, and the scaffold containing the most complete *Ghost* element is very short, at just 4918 bp in length. The remaining *Ghost* elements from aphid genomes were also found in short scaffolds (Additional file 2: Table S2), which appear to represent repeat rich regions where successive elements have inserted into each other. Discounting sequencing issues, *Ghost* MULEs may either represent an additional group of non-TIR MULEs (see below), or an old and no longer active MULE family where TIRs and TSDs have degraded. If the latter is the case, the intact DDE domains of these sequences may argue for their domestication for host purposes, otherwise it is unclear why other structural features should have been degraded but the DDE domain retained.

Elements in the *Spectre* clade were identified in the same host genomes as *Ghost* elements, with the exception of the black cherry aphid (*M. cerasi*) and the cnidarian hosts, and the addition of a number of arthropod hosts: three aphid species (greenbug *Schizaphis graminum*, Russian wheat aphid *Diuraphis noxia*, yellow sugarcane aphid *Sipha flava*), three true bugs (marmorated stink bug *Halyomorpha halys*, brown planthopper *Nilaparvata lugens*, silverleaf whitefly *Bemisia tabaci*), three beetles (mountain pine beetle *Dendroctonus ponderosae*, Colorado potato beetle *Leptinotarsa decemlineata*, red flour beetle *Tribolium castaneum*), and the Arizona bark scorpion (*Centruroides sculpturatus*). One complete *Spectre* element was identified, which originated from the genome of the green peach aphid (*M. persicae*). The complete element contains two ORFs, consisting of a C_2_H_2_ zinc finger domain and a transposase, and two perfect 74 bp TIRs, each flanked by an 8 bp TSD (please see annotation in Fig. 1b). The discovery of an intact transposase sequence and perfect TIRs and TSDs in *Spectre* suggests that this family is still active. Identification of a clade of closely related *Spectre* elements with short terminal branch lengths in the genome of the Colorado beetle (*L. decemlineata*), suggests this genome may contain a currently active group of elements, although we were unable to detect a fully complete *Spectre* sequence in this host.

Our analyses greatly expand the number of known invertebrate genomes that host MULE elements. Further, given the distribution of hosts observed in the *Ghost* and *Spectre* clades, we suggest that many more MULEs may await discovery in diverse arthropod genomes. Consequently, we anticipate that understanding of the host range and evolution of these families will increase considerably over coming years, as increasing numbers of invertebrate genomes are screened for transposons.

### Evolution and host range of MULEs

In line with previous studies, we identified MULE elements in the genomes of 6 diverse groups of eukaryotic life: animals, fungi, amoeba, stramenopiles, parabasilids, and plants. Such a wide diversity of distantly related hosts, alongside the observed branching pattern between host groups (Additional file 1), suggests a history of repeated horizontal transfer for MULEs. While once considered rare, horizontal transfer has recently become established as a strong force in shaping the distribution and evolution of TEs [33]. Our phylogeny, where high clade support unites hosts from diverse origins, suggests a tendency for certain MULEs to jump between hosts from distantly related branches of the tree of life, such as stramenopiles and platyhelminthes (group 16), stramenopiles, arthropoda and tunicata (group 20), and stramenopiles, plants and fungi (group 35) (Additional file 1). Remarkably, this last group includes MULEs from host species belonging to three different taxonomic kingdoms. A shared environment and close ecological associations may promote such groupings, for example, in groups 44 and 50, *Phytophthora* are widespread filamentous generalist plant pathogens that share an intimate relationship with their plant hosts [34], while *Chaetomium globossum* (group 35) is a widespread fungus that can colonise various habitats and cause opportunistic infections [35]. Similarly, a MULE previously reported from the genome of a polydnavirus (viruses injected into insect hosts by parasitic wasps to suppress host immunity) [24], clusters closely with other MULEs isolated from diverse invertebrate genomes (group 20, Additional file 1), including the jewel wasp (*Nasonia vitripennis*) and the red flour beetle (*Tribolium castaneum*), highlighting the potential role of viruses as vectors for the horizontal transmission of transposons. Several cases of the horizontal transfer of MULEs between plant hosts have also been suggested, including rice and other grasses in the genus *Setaria*, and rice and Old World bamboos [36]. Nevertheless, despite the close relationships shared between elements in the above mentioned host groups, and the apparent incongruence in observed host phylogenetic relationships, it is prudent to remain cautious when hypothesizing horizontal transfer events until a larger number of eukaryotic genomes have been screened for MULEs.

**Fig. 2:**
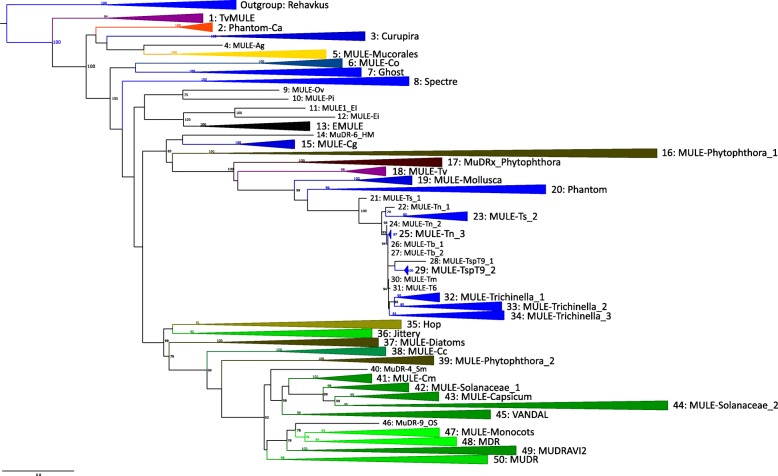
Schematic providing a summary of host associations for monophyletic MULE groups identified during phylogenetic analyses, which are illustrated as collapsed clades

There are few instances of MULEs from non-plant genomes in the more derived clades of MULE phylogeny (i.e. groups 36–49, Additional file 1). In the very few cases where non-plant MULE lineages do occur among more derived clades, they exist as isolated lineages, and are restricted to the plant parasite *Phytophthora* and one instance of the platyhelminth *Schmidtea* (groups 39 and 50, and the isolated taxon 40 MuDR-4_SM, Additional file 1). Thus, more derived MULE clades contain elements that are generally restricted to plant host genomes, perhaps reflecting a fundamental difference in the biochemistry of transposition for this group of elements in plants, or differing defence mechanisms against MULE transposons. In more basal clades, MULEs are typically distributed according to host group, either at a general level, or as radiations of elements restricted to individual host species or genera (e.g. group 1 and 12 *Trichomonas*, group 13 *Entamoeba*, group 19 Mollusca, group 21 to 34 *Trichinella,* Additional file 1). A cartoonised depiction of MULE phylogeny (Fig. 2), highlights the alternating associations of MULE elements among host groups across phylogeny, with the exception of more derived clades, which as mentioned above, contain elements almost exclusively originating from plant host genomes.

We find limited evidence for the existence of MULEs in chordates, with the exception of one sequence from the Pacific transparent sea squirt (*Ciona savigny*). The presence of MULES in the genomes of the Florida lancelet *Branchiostoma floridae* and horse *Equus caballus* were previously reported [24], yet while an amino acid alignment was provided for a FLYWCH zinc finger, none were provided for the transposases of these elements, and we were unable to detect evidence of MULE transposases in the nucleic acid sequences provided for these taxa, or during independent database searches. In Repbase, 9 reports of *MuDR* elements exist from vertebrate host genomes, 6 of which originate from the genome of the zebrafish *Danio rerio* and 3 from the genomes of placental mammals. However, of these 9 reports, only 3 are listed as autonomous elements (MuDR-1_DR, MuDR-2_DR, MuDR-3_DR) [37], their ORFs are extremely fragmented, and we were not able to detect a match between these sequences and MULE transposases. Moreover, we were not able to align the above mentioned protein sequences to our mutator alignment (Additional file 3), strongly suggesting that these supposedly vertebrate MULE sequences are in fact mis-identified. Consequently, the identity of the 4750 *MuDR* elements listed from the zebrafish and those from Eutherian genomes [38–41] require re-evaluation.

A striking observation from our analyses is that, with the exception of plant MULEs (which are mainly found in crops and other cultivated species), many of the genomes that contain MULE elements belong to parasitic/pathogenic species that either attack plants (e.g. insects, fungi and the Oomycete *Phytophthora*) or animals (e.g. diverse eukaryote groups including: *Schistosoma*, *Trichomonas, Nasonia, Lepeophtheirus, Trichinella, Candida*). A potential explanation for this pattern is a bias in sequencing effort directed towards parasitic species, due to their important impacts on health or agriculture. However, several alternative hypotheses may explain the potential increased load or activity of MULEs in parasite genomes [42–44]. For example, the genomes of parasitic *Phytophthora* and fungal pathogens are often considerably larger than those of non-parasitic stramenopiles and fungi, and their genome expansions are typically a consequence of increases in TE copy number [45]. Additionally, the hypothesis of “two-speed genomes” in filamentous plant pathogens suggests that repeat-rich regions of the genome evolve quickly, facilitating new regulatory functions and rearrangements that can enhance parasite adaptation to host defences, whereas gene-rich regions evolve more slowly [43]. Under this hypothesis, the success of MULEs in *Phytophthora* species (especially *P. infestans*), and the fungi *Puccinia graminis* and *Fusarium oxysporum* could be facilitated by a decreased stringency towards suppressing TE mobility. Similar processes may be applicable to parasitic insects, such as the peach potato aphid and Colorado potato beetle, which are globally important pests of crops [46, 47]. Selection for novel resistance mutations has been intense in many insect species over recent decades, following the extremely high exposures to insecticides experienced by many insect populations globally. Indeed, the role of successive TE insertions in promoting increased host insecticide resistance via the evolution of genomic novelty has been documented in *Drosophila* [48].

An interesting and diagnostic feature of MULEs is their unusually long TIRs [2]. As mentioned above, TIRs in MULEs vary greatly in length, and elements included in our analysis possess TIRs between 14 and 500 bp. Furthermore, this variation appears to be independent of phylogeny (Table 1, Additional file 1). It is not currently clear why such great variation exists in TIR length within and between MULE groups over phylogeny. TIRs contain promoters that trigger transcription of the transposase [49], and so are important for basic transposon functioning. However, it has been suggested that TIRs may be used by host genomes to distinguish between host genes and those of genomic parasites (given readthrough transcription of TEs resulting in fold-back at TIRs to form hairpin double stranded RNA structures) [50]. Indeed, long MULE TIRs are known to be associated with small interfering RNAs (siRNAs) and TIR loss is suggested to present a potential mechanism for escape from siRNA-based silencing [51]. Consequently, differing intensities of host-transposon co-evolutionary dynamics may provide a potential explanation for widespread variation observed in MULE TIR length, if this offers an alternative and less dramatic means of temporarily evading host detection than total loss of TIRs.

**Table 1:** A summary of the major characteristics of each MULE group identified in the phylogenetic analysis performed in this study (shadings as illustrated in Additional file 1)

Group No.	Group Name	Host phyla	TSD length	TIR length	ORF number	Zn finger type	Other motifs/ORFs
Outgroup	*Rehavkus*	Arthropoda and Annelida	9 bp	26–400 bp	1	PHD finger	Minisatellite in Cterm
1	*TvMULE*	Parabasalia	9–11 bp	20–38 bp	1	FLYWCH for 1	FAR1
2	*Phantom-Ca*	Ascomycota	9 bp	34 bp	1	–	–
3	*Curupira*	Platyhelminthes	9 bp	None	1	SWIM	–
4	MULE-Ag	Mucoromycota	amino acid sequence	–	–	–	–
5	*MULE-Mucorales*	Mucoromycota	10 bp	40 bp	1	SWIM	–
6	*MULE-Co*	Filozoa	Not indicated in RepBase	None	2	–	The second ORF is unknown
7	*Ghost* sub-group Cnidaria	Cnidaria	Not indicated in Repbase	177 bp and amino acid sequences	1	–	–
7	*Ghost*	Arthropoda	Not identified	None and amino acid sequences	1	SWIM	–
8	*Spectre*	Arthropoda	8 bp	76 bp	1	C2H2	–
9	*MULE-Ov*	Platyhelminth	amino acid sequences only	–	–	–	–
10	*MULE-Pi*	Oomycota	amino acid sequences only	–	–	–	–
11	*MULE1_EI*	Amoebozoa	9 bp	187 bp	1	–	
12	*MULE-Ei*	Amoebozoa	amino acid sequences only	–	–	–	
13	*EMULE*	Amoebozoa	amino acid sequences only	–	–	–	–
14	*MuDR-6_HM*	Cnidaria	Not indicated in RepBase	None	1	–	Ovarian Tumour Gene
15	*MULE-Cg*	Mollusca	Not indicated in RepBase	48 bp and 296 bp	1	–	–
16	MULE-Phytophthora_1 sub-group *Sm*	Platyhelminthes	9 bp	42–46 bp	1	SWIM and FLYWCH	–
16	*MULE-Phytophthora_1*	Oomycota	Not indicated in RepBase	None, except one with 71 bp	1 or 2	SWIM only in MuDR-5	SET for 14 members
17	*MuDRx-Phytophthora*	Oomycota	9 bp	40–80 bp	1	FLYWCH	–
18	*MULE-Tv*	Parabasalia	9 bp	100 bp and 31 bp	1	–	–
19	*MULE-Mollusca*	Mollusca	Not indicated in RepBase	None and amino acid sequences	1	–	–
20	*Phantom*	Platyhelminthes, Crustacea, Arthropoda, Tunicata and Ichnovirus	Not indicated/8-10 bp	18–613/None	1 or 2	None or FLYWCH or SWIM	Mini-satellite after the transposase ORF (aphids)
21–34	*MULE-Trichinella*	Annelida	Not indicated	None and amino acid sequences	1	–	–
35	*Hop* sub-group *MULE-Cglob*	Ascomycota	Not indicated in RepBase	51 bp and amino acid sequences	1	CCHC	–
35	sub-group *Mutyl*	Ascomycota	9–10 bp	22 bp	2	C_2_H_2_	Second ORF called MutB in antisens
35	sub-group *plants, Phytophthora, Puccinia*and Fungi	Magnoliophyta, Oomycota, Basidiomycota and Ascomycota	Not indicated in RepBase	30–220 bp and amino acid sequences	1	CCHC, C_2_H_2_ and SWIM	FAR1 in half the members
35	sub-group *Fungi*	Ascomycota	Not indicated in RepBase	85–99 bp and amino acid sequences	1 or 2	CCHC	–
36	*Jittery* sub-group *MULE-Cc*	Rhodophyta	Not indicated in RepBase	192 bp and 500 bp	1	SWIM	Various inserts, potentially host derived
36	sub-group Dicots and Monocots	Magnoliophyta	Not indicated/8 bp	39–292 bp, none and amino acid seq	1	SWIM or CCHC in Cterm	FAR1 in Nterm
37	*MULE-Diatoms*	Stramenopiles	No TSD and not indicated	123–124 bp and amino acid sequences	1	SWIM	–
38	*MULE-Cc*	Rhodophyta	9 bp and not indicated	244 and 640 bp	1	SWIM	–
39	*MULE-Phytophthora_2*	Oomycota	Not indicated in RepBase	81, 67 bp and amino acid sequences	1	SWIM and CCHC	–
40	*MuDR-4_Sm*	Platyhelminth	Not indicated in RepBase	53 bp	1	SWIM	–
41	*MULE-Cm*	Magnoliophyta	amino acid sequences only	–	–	–	–
42	*MULE-Solanaceae_1*	Magnoliophyta	9 bp and not indicated	None and amino acid sequences	1	SWIM	unknown ORF and PMD (antisens)
43	*MULE-Capsicum*	Magnoliophyta	amino acid sequences only	–	–	–	–
44	*MULE-Solanaceae_2*	Magnoliophyta, one Oomycota	9 bp and not indicated	None and amino acid sequences	–	–	–
45	*VANDAL*	Magnoliophyta	9 bp and not indicated	46–174 bp and amino acid sequences	1	SWIM and CCHC	PMD and/or DUF in half the members
46	*MuDR-9_OS*	Magnoliophyta	9 bp	45 bp	1	SWIM	PMD
47	*MULE-Monocots*	Magnoliophyta	Not indicated in RepBase	14–421 bp and amino acid sequences	1	SWIM or CCHC	PMD in most, sometimes antisense
48	*MDR*	Magnoliophyta	Not indicated/9 bp	32–373 bp and amino acid sequences	1	SWIM	PMD in most, sometimes antisense
49	*MUDRAVI2*	Magnoliophyta	Not indicated/9 bp	80–290 bp and amino acid sequences	1 or 2	SWIM or CCHC	PMD in most, often antisense
50	*MUDR*	Magnoliophyta	Not indicated/9–10 bp	30–320 bp and amino acid seq	1 or 2	SWIM (CCHC for MuDR-13B_Atr and MuDRB_PT)	PMD in antisense for most of them
50	sub-group *Phytophthora*	Oomycota	Not indicated in RepBase	40–55 bp	1	SWIM (C_3_HC_4_ ring finger for 37_Pi)	FAR1 in MULE-1_Pi and MuDR-2_Pso
50	sub-group *MUDRAVI*	Magnoliophyta	Not indicated/9 bp	63–447 bp and amino acid sequences	1	SWIM and C2H2 for two members	–

### MULE taxonomy

Progress in genome sequencing and the resultant emergence of new elements has outpaced evolutionary analyses of MULEs over recent years, resulting in several inconsistencies in MULE taxonomy. With reference to our phylogenetic analysis, which partition MULE diversity into a series of highly supported major MULE clades (Additional file 1), below we provide suggestions aimed at improving the clarity of MULE taxonomy.

*Phantom* is a structurally variable MULE family described from a diverse set of eukaryotic host taxa [24]. We included 13 canonical *Phantom* sequences [24] in our analysis. Our results strongly suggest that a supposed element from the genome of the pathogenic yeast *Candida albicans* (Additional file 1, group 2)*,* should be excluded from the *Phantom* family. The *C. albicans* element is the sister taxa to elements from the Platyhelminth *Schistozomas* and is separated from all other *Phantom* elements by several clades containing sequences from diverse host species. Remaining *Phantom* elements occur in 3 highly supported clades, broadly defined by host species, as follows: group 17, 23 *Phytophthora* elements; group 18, 32 *Trichomonas vaginalis* elements; group 20, 10 elements from various invertebrate hosts; and group 21, an isolated sequence from *Trichinella spiralis* (MULE-Ts_1). MULEs in group 17 from *Phytophthora* occur alongside many *MuDRx* elements from *P. infestans*. The original *MuDRx* element was detailed in a RepBase report in 2009 [52], ahead of the description of *Phantom* elements [24], so we suggest that it is preferable to maintain the name *MuDRx* for the family of elements represented by group 17. Elements in group 18 exist only from *T. vaginalis*, and since *Phantom* was described as a group of MULEs widely distributed in animals, it seems more appropriate to reserve the name for group 20 and reassign group 18 an alternative name, and so we refer to it here as MULE-Tv. *Phantom* elements in group 20 occur alongside “*Muta*” elements described from the tiger mosquito *Aedes aegypti* [21]. *Muta* elements were described recently [21], and so *Phantom* has priority as a name for this family of elements, and we suggest maintaining the status of group 20 as the *Phantom* family. Given that group 21 represents a single MULE sequence from *T. spiralis* we name it MULE-Ts_1.

Issues relating to the nomenclature of several plant MULE clades are apparent. Jittery, described in maize, occurs together with a *MUJITOS2* element from rice (*Oryza sativa*), a *MUMET1* element from barrelclover (*Medicago truncatula*), a *MUTRIM1* element from einkorn wheat (*Triticum monococcum*) and multiple *MuDR* elements from both monocotyledonous and dicotyledonous host plant genomes (group 36, Additional file 1). With reference to MULE phylogeny (Additional file 1), it is apparent that these lineages occur as part of a larger, highly-supported clade of plant MULEs (group 36) including *Jittery*, which has the unusual transposition behaviour of apparent excision without reinsertion [53].

*MUDRAVI* elements constitute a polyphyletic group represented by clades including the elements *MUDRAVI1* and *MUDRAVI2* (groups 49 and 50 respectively, Additional file 1). The *MUDRAVI2* group (group 49) contains elements extracted from the grape vine (*Vitis vinifera*) genome, and many sequences from dicotyledonous plant genomes, whereas the *MUDR* group (group 50), contains elements extracted from a wide range of different host plant species, including *MUDRAVI1* from grapevine, tobacco (*Nicotiana sylvestris*), soybean (*Glycine max*), a diploid cotton (*Gossypium raimondii*), one sequence from white jute (*Chorcorus capsularis*), *ATMU* from Arabidopsis, *SHAMUDRA* from the barrelclover, and several sequences from cereals, among them *OSMU* and *RMU1A23* from rice. We named this clade *MUDR* (from maize) as it is the oldest element with a specific name in this group.

We find support for the existence of a clade containing multiple *VANDAL* elements (group 45, Additional file 1). *VANDAL* elements are notable TEs since they are apparently autonomous but appear to either totally lack or have short degenerate terminal inverted repeats (TIRs), and were the first non-TIR autonomous DNA TEs described in eukaryotes [51]. A transposition mechanism that does not require TIRs may provide an efficient means to avoid host genomic defences against TE mobility [10]. The anti-silencing protein VANC may be involved in such a mechanism, as it induces hypomethylation of these elements, facilitating their transcription [54]. The original *VANDAL* element was described from *Arabidopsis* [51], but it occurs together with elements from other Brassicaceae plant genomes (group 45, Additional file 1 and Table 1), some of which do contain TIRs, suggesting a potential later evolutionary switch to a non-TIR based mechanism of transposition.

### Origin of MULES and relationship to Rehavkus elements

If the MULE superfamily is considered to exclude *Rehavkus* elements, the host of the earliest branching MULE clade is the parabasalid excavate *Trichomonas vaginalis*, while the next earliest branching clade includes elements from the platyhelminth *Schmidtea mediterranea* and the oomycote stramenopile *Phytophthora infestans*. However, if *Rehavkus* is considered to be a MULE family, a metazoan origin for the entire group is more plausible, given that *Rehavkus* is restricted to insect and annelid worm hosts (Additional file 1, Table 1). To date, very few *Rehavkus* elements have been identified and known elements are described in Repbase only [55]. The structure of *Rehavkus* elements is similar to that of MULEs, except for the presence of a mini-satellite sequence prior to the 3′ TIR and the possession of a PHD finger in the transposase domain (Additional file 4: Table S1). In a broad-scale phylogenetic analysis examining the phylogenetic relationships of the catalytic domain of all eukaryotic cut-and-paste transposase superfamilies, Rehavkus elements were lumped together with MULEs, on the basis of several conserved residues in the DDE domain, similar length TIRs, and especially their shared long TIRs which are a particular feature of some MULEs [26].

## Conclusions

MULEs are important TEs due to their high activity rates, bias toward inserting close to genes, and applications in genetic research [2, 7, 56]. MULEs are also notable for their structural variability, including their frequent take up of foreign DNA and highly variable TIRs, and because of the unusual transposition behaviour displayed by members including *VANDAL* [2, 53, 57]. Recent years have seen a great increase in the diversity of MULE elements and their known hosts, particularly outside of plants, and evidence now exists that is suggestive of a history of frequent host switching across phylogeny [21, 22]. Consequently, MULEs offer considerable promise as a model DNA TE system for varied research questions in host-transposon co-evolutionary interactions.

Our phylogenetic analyses divide MULE diversity into a number of major families, and we identify several new MULE clades, greatly expanding knowledge of elements from arthropod hosts in particular. Our results suggest that MULEs are more prevalent outside of plant hosts, which are traditionally considered as their focal host group. Due to the lack of recent evolutionary analyses, our findings significantly increase understanding of MULE diversity and evolution. Given our findings, it is likely that many more MULE elements await discovery, both in plants and in their other hosts. Hence, to facilitate future research on MULEs, we propose an updated taxonomic context for partitioning MULE diversity.

Many fundamental questions concerning MULE biology remain to be addressed. These include: (i) Are MULEs over represented in the genomes of parasitic species, or is this an artifact based on sequencing bias? (ii) What is the biochemical and evolutionary significance of the apparent hard division between MULEs in plants and MULEs in other host groups? (iii) To what extent is the apparent pattern of horizontal transmission across highly divergent host groups a real feature of MULE biology, which is upheld given increased taxonomic sampling? (iv) Which vectors represent the main vehicles for the horizontal transfer of MULEs, and particularly, do viruses represent a common route of transfer between host taxa? (v) What is the evolutionary significance of the often extremely long and highly variable TIRs of MULEs? (vi) Are MULEs really absent from vertebrate genomes, or have they been excluded from hosts beyond the shallower clades of chordate phylogeny? (vii) How can the extraction of phylogenetic information from MULEs be improved, in order to maximise phylogenetic signal and minimise noise, and thus enable elucidation of the deeper nodes of MULE phylogeny? Tackling these questions will lead to significant improvements in our understanding of MULE biology and the forces that shape host-transposon interactions more widely.

## Methods

### Retrieval of MULE elements

We discovered an unknown TE sequence in the genome of the green peach aphid *Myzus persicae* which we name *Spectre*. *Spectre* shared features in common with *Mutator-*like elements (MULEs), with 8 bp TSDs and 74 bp TIRs, and it clustered firmly in the middle of known MULE phylogeny. Consequently, *Spectre* was used to search genomes present in the online aphid genome database AphidBase, using BLASTn and BLASTx to identify related sequences. The following aphid genome releases were searched (representing the most recent releases in each case at the time of searching): *Acyrtosiphon pisum*, genome v2, *Aphis glycines* genome, *Diuraphis noxia* genome 1.0, *Myzus cerasi* genome v1.1, *Myzus persicae* Clone G006 assembly v2 and *Ropalosiphum padi* genome v1.0. These searches revealed that additional elements closely related to *Spectre* were present in the *M. persicae* genome, the *Acyrtosiphon pisum* genome, and in the *M. cerasi* genome. After searching available aphid databases, we performed an extensive search for elements similar to *Spectre* using BLASTn and BLASTx queries on NCBI GenBank, retaining all hits with ≥50% identity that were ≥ 50% of the length of the query sequence.

To collect known MULEs for a comprehensive bioinformatic review of the *Mutator* superfamily, we performed text searches of NCBI GenBank using common abbreviations for MULEs (e.g. MuDR, Mutator, Mule, Mu), retaining any elements containing complete or near complete transposase domains. Additionally, we downloaded amino acid sequences for all autonomous eukaryote MULEs available in Repbase [58]. We also downloaded sequences for MULE transposons described in the supplementary information of relevant publications and elements corresponding to these articles from Genbank [9, 18, 21, 22, 24]. Lastly, elements were included from another article on request [26]. After assembling a wide range of MULE sequences, we performed a phylogenetic analysis and identified a large number of highly supported monophyletic MULE clades. Using one sequence per clade as a query in turn, we then performed successive BLASTn, BLASTx, tBLASTn, and PSI-BLAST searches on NCBI GenBank to identify further MULE sequences, as well as BLASTx and tBLASTx searches on two other databases: Ensembl Genomes [59] and FlyBase [60].

In this study we focus analyses on MULE elements from eukaryote genomes. Insertion sequence elements (IS elements) in prokaryotic genomes with similarity to MULE transposases are known (i.e. the IS256 group), however, these are considered distantly related to eukaryotic MULEs, which form a well-supported monophyletic group to the exclusion of MULE-like insertion sequences in phylogenetic analyses [18, 22].

### Annotation of retrieved elements

Retrieved elements were annotated with Artemis software [61], using the ORFfinder and BLASTx tools on NCBI for conserved domains, and the Palindrome analyser tool of DNA Analyser to find TIRs [62]. Motifs such as zinc finger domains were identified using MOTIF (https://www.genome.jp/tools/motif/) or the BLASTp option of ORFfinder.

### Phylogenetic analyses

In total, amino acid sequences for 1631 MULEs were collected (for details see Table 1 and Additional file 4: Table S1). For phylogenetic analyses we focussed on the transposase domain, since this is the only highly conserved region of DNA TEs. Transposase domains were aligned using Muscle [63], using the profile alignment option and the DDE domain alignment of Yuan & Wessler 2011 [26], as a basis for alignment. The alignment was manually curated using MEGA v7 [64] and Geneious v11.1.4 [65]. We used members of the *Rehavkus* TE group as an outgroup to root our phylogeny, since these are closely related to canonical MULEs [26]. Some MULE elements from Repbase did not align to other sequences, and were consequently not considered as MULE elements and were not included in phylogenetic analyses (see Additional file 3).

To infer the evolutionary history of MULEs, we used PartitionFinder2 [66] to identify the best fitting amino acid evolution model for our alignment, followed by a maximum-likelihood phylogenetic analysis with IQ-tree [67]. The selected model of amino acid evolution was VT + G and we performed 1000 bootstrap repetitions [68].

Following phylogenetic analysis, ClusterPicker [29] was used to infer phylogenetic clusters based on bootstrap support values and genetic distance. Both the initial and main bootstrap support thresholds were specified as 80%. We examined a range of genetic distance thresholds from 1.5 to 4.5% (covering the range of suggested settings in ClusterPicker [29]), at an interval of 0.1%. Following this, a graph of the relationship between the number of clusters inferred and the applied genetic distance threshold was examined (Additional file 5). With reference to group delineation, there is a first major decrease in the number of identified clades when applying a 3.6% sequence distance threshold. There is a subsequent major decrease at a threshold of 3.8%, and a final major decrease at a threshold of 4.2%, before the pattern plateaus out at 10 very large inferred groups. We chose to adopt the 3.6% threshold since this delineates clades at a relatively inclusive level (avoiding excessive splitting), and is most consistent with previously identified clades and patterns in TE host range. However, we include trees for each threshold at 0.1% increments between 2.6–4.2% for reference (Additional file 6).

MULE taxa in Additional file 1 were colour labelled according to host taxonomy (at the level of phyla or similar) in FigTree v1.4 (tree.bio.ed.ac.uk/software/figtree/). Clade names correspond to the host taxonomic group that they belong to, or to the first described MULE in the literature for taxa including more than one element (e.g. *Jittery*, group 36).

Sequences of the newly identified elements in the *Ghost* and *Spectre* clades have been deposited in GenBank, with the following accession numbers: *Ghost*: MH937730 and *Spectre*: MH937731. Records for these elements will also be deposited into the online repeat database RepBase.

## Additional files

Additional file 1: Phylogenetic tree of the amino-acid DDE transposase domain of 1631 autonomous *Mutator*-like elements. The tree results from a phylogenetic analysis using maximum likelihood inference, with 1000 bootstrap repetitions. Clade support values above 0.75 are indicated adjacent to each clade. Clades are divided into groups, as indicated by alternate shading, with a corresponding clade name and number to the right. For each clade (except eight groups for which we only recovered the amino acid transposase domain), a schematic summarising the structure of the TEs contained within each group is illustrated, with structural features represented by different coloured rectangles (please see the accompanying key). Elements are named according to their Repbase or Genbank ID, or according to the name provided in the article describing them. The host genome for each element is indicated to the right hand side of its ID, and labels are coloured broadly according to the taxonomic kingdom that the species belongs to: blue for Metazoa, purple for Excavates (Parabasalids), taupe for Oomycetes and Diatoms (Stramenopiles), black for Amoebozoa, orange/yellow for Fungi, and green for Plantae. Family-level groupings for MULE clades consisting of ≥2 elements are indicated with right curly braces. When more than one schematics represent the structure of the elements within a clade, dotted lines indicate which elements the schematic depicts. The alignment used for this phylogenetic analysis is provided in Additional file 7. (SVG 6820 kb)

Additional file 2: **Table S2.** Genomic features and characteristics of each *Ghost*, *Spectre* and new aphid *Phantom* elements identified from the aphid genomes analysed. (XLSX 13 kb)

Additional file 3: An amino acid alignment of elements that were removed from the phylogenetic analysis as a consequence of their lack of alignment with other MULE DDE domains. (FASTA 94 kb)

Additional file 4: **Table S1.** Information on the different clusters indicated in Additional file 1. (XLSX 41 kb)

Additional file 5: A graph of the number of assigned clusters according to the thresholds applied in each ClusterPicker analysis. (JPG 122 kb)

Additional file 6: Output trees from ClusterPicker with each inferred cluster coloured alternately, at the genetic distance thresholds of: 2.6, 3.6, and 4.2%. (ZIP 170 kb)

Additional file 7: The amino acid alignment used to perform our phylogenetic analysis. (FASTA 6699 kb)

## Abbreviations

MULEs: *Mutator*-like elements
ORF: Open reading frame
siRNAs: Small interfering RNAs
TEs: Transposable elements
TIRs: Terminal inverted repeats
TSD: Target site duplication
