# Supplementary figures and images for: Evolution of Mutator transposable elements across eukaryotic diversity
Source: Mob DNA. 2019 Mar 21;10:12. doi: 10.1186/s13100-019-0153-8 (PMC6446971; doi:10.1186/s13100-019-0153-8)

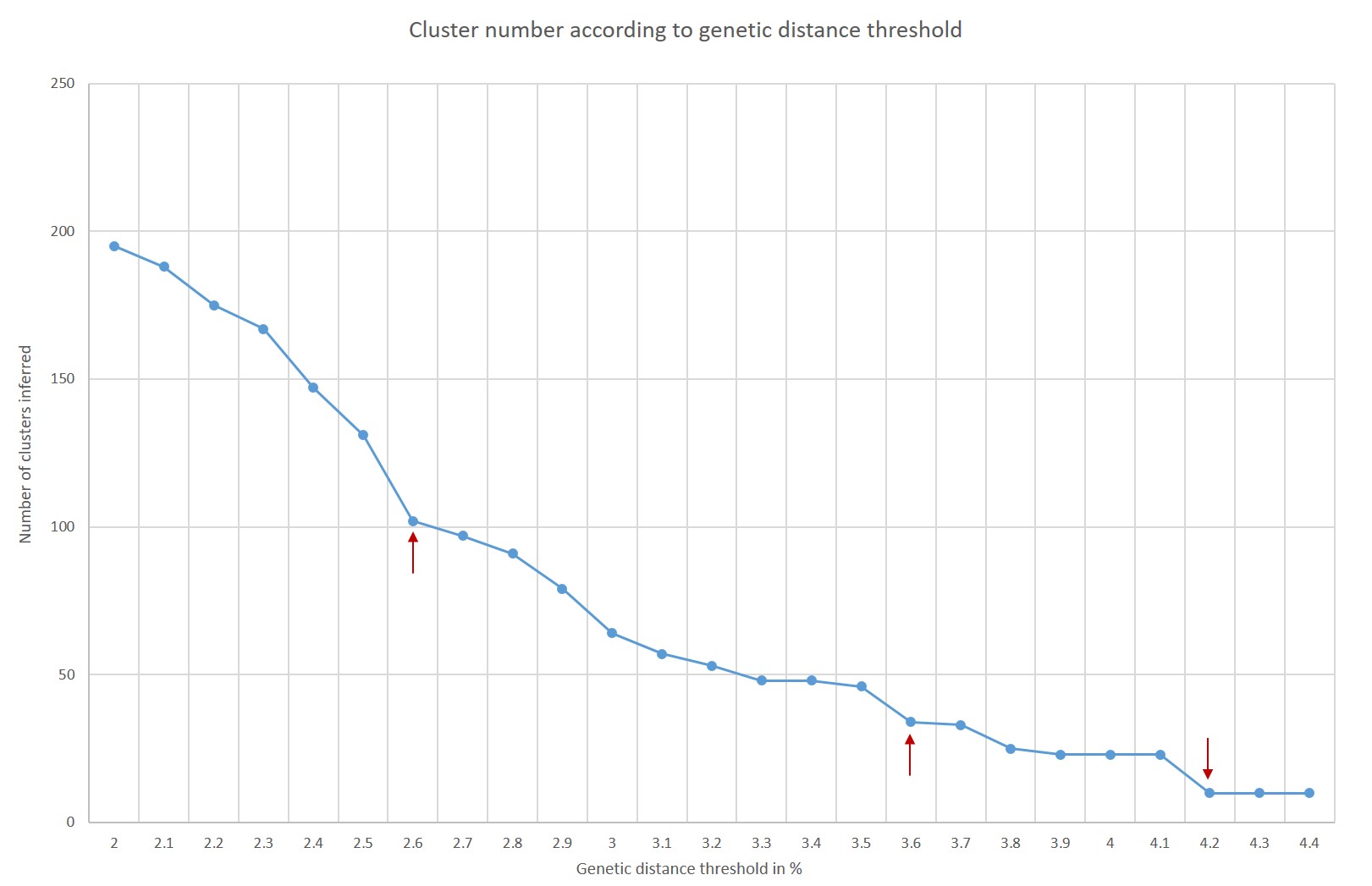

Supplement: Supplementary file 5 — A graph of the number of assigned clusters according to the thresholds applied in each ClusterPicker analysis. (JPG 122 kb) [file 13100_2019_153_MOESM5_ESM.jpg]
